# The Management of Primary Hyperaldosteronism in a Poor Technology Environment

**DOI:** 10.1155/2021/6672052

**Published:** 2021-05-10

**Authors:** Jean Sossa, Dedjinnin Josue Georges Avakoudjo, Dodji Magloire Ines Yevi, Lionelle Fanou, Gilles Natchagande, Michel Michael Agounkpe, Fred Hodonou, Yao Felicien Hounto, Felix Atadokpede

**Affiliations:** ^1^Service d'Urologie, Hopital d'Instruction des Armees Centre Hospitalier Universitaire (HIA-CHU), Cotonou, Benin; ^2^Clinique Universitaire d'Urologie-Andrologie, Centre National Hospitalier et Universitaire Hubert Koutoucou Maga (CNHU-HKM), Cotonou, Benin

## Abstract

We report a case of Conn's adenoma in a 35-year-old female successfully managed in a poor hospital technology environment.

## 1. Introduction

The primary hyperaldosteronism is the most frequent cause of secondary hypertension [1–4]. Diagnosis and management of the condition are often delicate. We report one case of a right aldosterone-producing adrenal adenoma or Conn's adenoma.

## 2. Case Presentation

**Figure 1:**
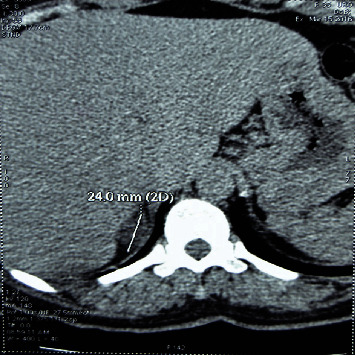
Right adrenal mass in the women.

**Figure 2:**
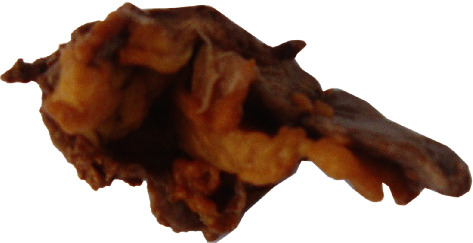
The right adrenal mass after adrenalectomy.

The patient was a nulliparous 35-year-old female with a 3-year long history of recurrent dizziness and convulsions. She described a monthly frequency of up to 3 bouts of convulsions. Nine months before she consulted in our institution, a hypokalemia was discovered and treated with oral supplementation of potassium, one 600-milligram tablet daily. As the bouts of convulsions went more and more frequent, potassium tablet intake was progressively increased up to 6 tablets daily. Still, the bouts of convulsions which were said to have drastically decreased did not disappear. But the woman could not afford an evacuation abroad as her previous care provider advised her to do. Rather she resorted to our institution. On exam, a 172/110 millimeters of mercury high blood pressure was discovered. The cardiologist confirmed the hypertension and started treating it with amlodipine 10 mg daily. Potassium level was low, 2.10 milliequivalents per liter. Adrenal hormones' workup on a venous blood sampled at 09:15 am after the patient rested supine on a couch for more than 1 hour showed an elevated aldosterone level of 2.496 × 10^−9^ mole per liter, i.e., 4.5 times the laboratory's 5.55 × 10^−10^ moles per liter threshold. Were normal the levels of renin (1.03 × 10^−2^ International Unit per liter), cortisol (1.85 × 10^−7^ mole per liter), metanephrine (2.7 × 10^−10^ moles per liter), and normetanephrine (9.4 × 10^−10^ moles per liter). The elevated aldosterone to renin ratio (242, i.e., 3.78 times the laboratory's threshold 64) was indicative of a primary hyperaldosteronism. An abdominal computed tomography revealed a 24 mm diameter right adrenal mass in the woman (Figure 1). The nodule was hypodense, homogeneous, regularly limited, and exhibited a 65% washout rate. The diagnosis of an aldosterone-producing adenoma was quite evident. An electrocardiogram was performed which showed a left auricular hypertrophy and an overloaded left ventricle. Plasma levels of creatinine, glucose, and hemoglobin were normal, respectively, 7 milligrams per liter, 1.09 grams per liter, and 10 grams per deciliter. We performed a right adrenalectomy through a right subcostal incision under general anesthesia (Figure 2). We mobilized leftward the right hepatic flexure of the colon and entered into the renal fascia. Downward reclination of the kidney enabled us to dissect and remove the nodular right adrenal gland without any bleeding incident nor any injury to adjacent organs. The anesthesiologist corrected the potassium level which became 4.9 milliequivalents per liter on the eve of the surgery. The patient resumed walking on the second postoperative day. Amlodipine was discontinued on the third postoperative day. Potassium level was monitored daily. The patient was discharged from hospital on the seventh postoperative day with potassium intake completely stopped. Stitches were removed on the tenth postoperative day. One month after the operation, there was no complaint, the potassium level was 4.4 milliequivalents per liter, and the blood pressure was 130/90 millimeters of mercury. At the end of the sixteenth postoperative months, the patient was still good with a normal blood pressure = 127/96 millimeters of mercury. Four years after surgery, the potassium level was 4.5 milliequivalents per liter with no complaint.

## 3. Discussion

A deep hypokalemia alongside a hypertension and bouts of convulsions allowed the diagnosis of Conn's adenoma in our patient. As it abnormally produces aldosterone in excess, a Conn's adenoma triggers a sodium retention, a potassium depletion, and a reduction of renin production, leading to hypokalemia and hypertension. Nevertheless an aldosterone-producing adrenal adenoma may be hypokalemic or less commonly normal potassium leveled [5]. Also, other metabolic disorders such as a hypocalcemia may be associated with hyperaldosteronism [6]. The computed tomographic detection of a right adrenal mass in the woman has been crucial to the diagnosis and to the surgical treatment decision-making. In fact, primary hyperaldosteronism can come from other adrenal disorders such as a bilateral aldosterone-producing adrenal adenoma, a unilateral or bilateral adrenal hyperplasia, a unilateral aldosterone-producing adenoma associated with a contralateral adrenal hyperplasia, or even an aldosterone-producing adrenocortical carcinoma [7–10]. Pathological examination of the specimen has confirmed the presence of a Conn's adenoma, thereby excluding any primary hyperaldosteronism associated with a Cushing adenoma or a unilateral estrogen-producing adrenal adenoma [11–13].

We have performed an open right adrenalectomy in our patient. Surgery is the standard treatment for a unilateral aldosterone-producing adenoma. An adrenalectomy is best performed under laparoscopy or retroperitoneal endoscopy. The hospital sojourn is shorter after those mini-invasive procedures: the mean inward stay ranges from 1.1 to 2.6 days. [14, 15]. Obviously the little scars at the laparoscopic ports' sites are much more aesthetic than the one left by an open surgical incision. Anyway, the removal of the adenoma is the one that will suppress the hyperaldosteronism and its impacts, not the type of surgery. However, the clinician must inform the patient that the adrenalectomy does not always correct the hyperaldosteronism-driven hypertension: the older the patient is at surgery or the longer his hypertension has lasted before surgery, the less likely the total resolution of the hypertension [16–21]. Adrenal sparing surgery is feasible for bilateral aldosterone-producing adenoma [7].

The detection of aldosterone-producing adrenal adenoma amenable to surgery is not easy. Once primary hyperaldosteronism is diagnosed, selective adrenal vein sampling is the gold standard method to determine which one of the left and right adrenal glands is responsible for the disorder [7, 22–27]. Some researchers are assessing diagnostic tools less invasive than adrenal vein sampling such as the 18 oxo-cortisol plasma level [28]. Adrenal vein sampling is costly and requires special skill and equipment that are not yet available in Benin. Nevertheless, in patients less than 35 years old with evident hypokalemia and unilateral adrenal adenoma greater than 10 mm diameter, adrenal vein sampling is not mandatory [29, 30]: the clinician can rely on the aldosterone to renin ratio coupled with imaging. Best imaging for adrenals are computed tomography (sensitivity = 84.3%) and magnetic resonance imaging (sensitivity = 97%, specificity = 90%) [29]. Sometimes imaging can be normal, and reimaging months later may detect an adrenal adenoma [31].

## 4. Conclusion

In limited means' working conditions, a careful combination of aldosterone to renin ratio and imaging can help to successfully manage a primary hyperaldosteronism.

## Data Availability

Data underlying this report are available in the archives of the HOPITAL D'INSTRUCTION DES ARMEES CHU Cotonou and are reachable via the corresponding author.
